# Assessment of pulmonary mechanics in mechanical ventilation

**DOI:** 10.1186/s13054-017-1733-y

**Published:** 2017-06-19

**Authors:** Federico Gordo, Beatriz Lobo-Valbuena

**Affiliations:** 1 Intensive Care Unit, Hospital Universitario del Henares, Avenida María de Curie s/n, 28822 Coslada, Madrid Spain; 2 Universidad Francisco de Vitoria, Carretera Pozuelo a Majadahonda, 28223 Pozuelo de Alarcón, Madrid Spain

We read with great interest the article published by Chen et al. [1] regarding a systematic assessment of respiratory mechanics in patients with ARDS to determine ventilation parameters and the optimal mode. They used basic monitoring along with the placement of an esophageal balloon catheter to measure transpulmonary pressure. We believe that monitoring pulmonary mechanics may be useful, not only in these patients, but in the whole ICU population, allowing individualized ventilation. This may also be achieved by other advanced monitoring systems, such as electrical impedance tomography [2].

However, we fail to understand how the changes in PEEP cause changes in pulmonary recruitment and oxygenation without modifying—according to the authors—the physiological dead space.

Furthermore, the authors report how the monitoring of these patients led to changes in the mechanical ventilation mode, leading to a volume-controlled mode. Nonetheless, in their study they do not include the respiratory mechanics data on which they based these changes. In the discussion they refer to three factors—the control of the tidal volume (Vt), better monitoring of the pulmonary mechanics, and the possibility to place an inspiratory pause—that, in our opinion, are neither conclusive nor exclusive of the volume-controlled modes [3]. In fact, studies have suggested that better distribution of Vt can be achieved using pressure-controlled modes, generating a more homogenous ventilation and enabling an increase in the inspiratory time. Moreover, Villar et al. [4] have demonstrated less pulmonary damage using pressure-control ventilation modes, regardless of the selected Vt and PEEP.

We do believe that the generalization of systematic respiratory mechanics assessment is useful to improve ventilation in ARDS patients, but these procedures need to be standardized. Some authors argue that these tests do not take into account the heterogeneity of lung injury and prefer other approaches to adjust the ventilation of these patients [5].

## Abbreviations

ARDS: Acute respiratory distress syndrome
ICU: Intensive care unit
PEEP: Positive end-expiratory pressure
Vt: Tidal volume
